# ALCOHOLIC VS. NON-ALCOHOLIC CHRONIC PANCREATITIS: SURGEONS’ PERSPECTIVE FROM A TERTIARY CENTRE IN INDIA

**DOI:** 10.1590/0102-672020210002e1595

**Published:** 2021-10-18

**Authors:** Koustav JANA, Sukanta RAY, Roby DAS, Dilip KUMAR, Tuhin S MANDAL, Somak DAS

**Affiliations:** 1 Department of Surgical Gastroenterology, SSKM Hospital and IPGMER, Kolkata, West Bengal-700020, India

**Keywords:** Chronic pancreatitis, Alcoholic, Frey procedure, Quality of life, Pancreatite crônica, Alcoolismo, Procedimento de Frey, Qualidade de vida

## Abstract

***Background*:**

Although alcohol is the most common cause for chronic pancreatitis worldwide, idiopathic type is prevalent in India. Natural history and disease progression are different between these two groups. There is paucity of data comparing surgical outcome and quality of life in these patients.

***Aim*:**

To evaluate clinical features, surgical outcome and quality of life between these two groups of patients.

***Method*:**

All patients with chronic pancreatitis who underwent surgery were prospectively reviewed.

***Results*:**

From 98 patients, 42 were alcoholic. Number of male and the mean age at the time of operation was significantly more in alcoholic patients. Smoking, preoperative hospital admission rate and the prevalence of local complications like inflammatory pancreatic head mass, biliary stricture and left sided portal hypertension were distinctly more common in alcoholic group. Frey procedure was required more commonly in alcoholic group. Mean postoperative hospital stay and overall postoperative complication rate were comparable between the two groups. Over a median follow up of 18 months there was significant improvement in quality of life and pain score in both the groups. Improvement of physical functioning score at follow-up was significantly more in alcoholic group but the requirement for analgesic medications were significantly more in alcoholic group. However, appetite loss was more perceived by non-alcoholic group.

***Conclusion*:**

Alcoholic chronic pancreatitis presents with more local complications associated with chronic pancreatitis. Frey procedure is a safe and well accepted surgery in this group. Though they required more analgesic requirement in short term follow up, other aspects of quality of life are similar to non-alcoholic group.

## RESUMO

***Racional*:**

Embora o álcool seja a causa mais comum de pancreatite crônica em todo o mundo, a forma idiopática é prevalente na Índia. A história natural e a progressão da doença são diferentes entre esses dois grupos. Há escassez de dados comparando o resultado cirúrgico e a qualidade de vida entre eles.

***Objetivo*:**

Avaliar as características clínicas, o resultado cirúrgico e a qualidade de vida entre esses dois grupos de pacientes.

***Método*:**

Todos os pacientes com pancreatite crônica operados foram revisados ​​retrospectivamente.

***Resultados*:**

Do total de 98 pacientes, 42 eram alcoolistas. O número de homens e a idade média no momento da operação foi significativamente maior nos alcoolistas. Tabagismo, taxa de internação pré-operatória e prevalência de complicações locais como massa inflamatória da cabeça do pâncreas, estenose biliar e hipertensão portal do lado esquerdo foram distintamente mais comuns no grupo de alcoolistas e o procedimento de Frey foi exigido mais comumente neste grupo. A média de internação pós-operatória e a taxa geral de complicações pós-operatórias foram comparáveis ​​entre os dois grupos. Ao longo de acompanhamento médio de 18 meses houve melhora significativa na qualidade de vida e pontuação de dor em ambos os grupos. A melhora no escore de funcionamento físico foi significativamente maior no grupo de alcoolistas, mas a necessidade de medicamentos analgésicos foi significativamente maior nos alcoolistas. No entanto, a perda de apetite foi mais percebida pelo grupo não alcoólico.

***Conclusão*:**

A pancreatite crônica alcoólica se apresenta com mais complicações locais associadas à pancreatite crônica. O procedimento de Frey é operação segura e bem aceita neste grupo. Embora exigissem mais necessidade de analgésicos no acompanhamento em curto prazo, outros aspectos da qualidade de vida são semelhantes ao grupo não alcoólico.

## INTRODUCTION

Chronic pancreatitis (CP) is a progressive inflammatory disease leading to irreversible damage of the pancreas. The most common etiology in West is chronic alcohol intake whereas idiopathic pancreatitis (or tropical pancreatitis) is reported to be most common etiology in India and China, accounting for approximately 70% of all patients^13^. Recent few papers have suggested alcoholism as a dominant cause of it in India^15^ ^,^ ^22^. Though genetic study and clinical features have suggested that tropical pancreatitis is different from idiopathic chronic pancreatitis^12^, one thing is common in both idiopathic and tropical pancreatitis: absence of alcohol intake. Natural history of alcoholic pancreatitis is different from non-alcoholic^2^. One recent study has shown the different genetic pattern for alcoholic chronic pancreatitis than other chronic pancreatitis^28^. In this part of India where genetic analysis is not prevalent widely, etiology of CP can be broadly classified as alcoholic and nonalcoholic. 

Pain is the most distressing symptom and most of the patients seek medical advice for disabling pain. Since chronic pancreatitis is a benign non-curable disease with a protracted natural course, pain relief as the successful outcome of the disease reflects only one aspect of the multidimensional outcome. As result, health-related quality of life as subjectively perceived by the patient, is becoming a major issue in the evaluation of any therapeutic intervention, mainly in patients with chronic diseases. In the current step-up approach, surgery is regarded as a last resort when other modalities fail. Several studies^3^ ^,^ ^4^ ^,^ ^17^ ^,^ ^18^ ^,^ ^24^ ^,^ ^25^ have shown that surgery for CP leads to pain relief and substantial improvement of health related quality of life in long-term follow up. Most these studies evaluate the nature of surgery and their long-term effect on the course of the disease. However, the quality of life in any alcoholics is quite different from healthy individuals or even from non-alcoholic patients. Several psychological and social factors interplay to determine the quality of life in alcohol dependents^23^. As earlier stated the natural history of alcoholic chronic pancreatitis is different from non-alcoholic^2^, treatment modalities and outcome may differ in long-term follow up. 

The aim of this study was to evaluate clinical features, surgical outcome and quality of life after surgery in alcoholic and nonalcoholic chronic pancreatitis.

## METHODS

This prospective observational analysis was performed in the Department of Surgical Gastroenterology, School of Digestive and Liver Diseases, Seth Sukhlal Karnani Memorial Hospital and the Institute of Post-Graduate Medical Education&Research, Kolkata, India. Between October 2016 and October 2019 all patients who underwent surgery for chronic pancreatitis were included. Patients diagnosed with malignant disease, h/o acute illness within four weeks and inability to understand the questionnaire were excluded from the study. All patients had a detailed history and clinical examination. The diagnosis of chronic pancreatitis was based on typical history of abdominal pain and pancreatic parenchymal/ductal calcification and/or dilatation of the main pancreatic duct on imaging. Alcoholic CP was diagnosed in presence of daily alcohol intake of >80 g/day alcohol for at least five years^1^.Other investigations included complete hemogram, liver function test, and renal function test. Upper gastrointestinal endoscopy was suggested in suspicion of portal hypertension.

### Operative procedures

Decision and type of surgery were finalised after multidisciplinary meeting between gastroenterologist, radiologist and primary surgeon. Only pancreatic ductal drainage (longitudinal pancreaticojejunostomy - LPJ) was performed in presence of dilated duct caused by stricture or stone, without an inflammatory head mass. A jejunal Roux loop was anastomosed side to side to whole length of pancreas from tail to head in single layer of continuous suture (3-0 or 4-0 polypropylene). We kept the length of the Roux limb around 60 cm as it could be used later for biliary bypass if the patient develops biliary stricture in future. Drainage procedure combined with a local resection as described by Frey and Smith^11^ was performed in presence of an inflammatory large head mass (>4 cm or more). However, simultaneous bilioenteric bypass was performed with the same loop in presence of obstructive jaundice or common bile duct stent. Izbicki procedure was performed in small duct disease (MPD diameter ≤3 mm) as described by Izbicki and colleagues^18^. Reconstruction was similar to LPJ. Pancreaticoduodenectomy was performed only in case of severe duodenal obstruction or malignancy could not be ruled out preoperatively. Distal pancreatectomy with or without splenectomy was performed in case of extensive disease at distal body or tail region. However, patients who were found to have malignancy after histopathological examination were excluded from the study.

### Data collection and follow up

Clinicopathological and operative data were collected from our prospectively maintained database. Postoperative complications were assessed according to Clavien-Dindo classification^8^ and postoperative mortality was defined as death within 90 days after the operation. Postoperative pancreatic fistulas, delayed gastric emptying and postoperative haemorrhage were diagnosed and classified based on International Study Group of Pancreatic Surgery criteria^6^ ^,^ ^26^ ^,^ ^27^. The patients were followed in the outpatient department. Follow up protocol in our department is every three months for the first two years, then six monthly for next three years, then annually for rest of the life. In the follow up, following parameters were recorded: body weight, pain, analgesic requirement, need for enzyme supplementation, need for hospitalization and blood sugar (both fasting and postprandial). Diabetes mellitus or endocrine insufficiency was defined if fasting blood sugar was more than 126 mg/dl and serum glycosylated hemoglobin was more than 6.5%. Pancreatic exocrine insufficiency was considered in presence of steatorrhoea and or the need to take pancreatic enzyme supplementation. Fecal elastase estimation was not available in our institution to assess pancreatic exocrine function. 

### Quality of life assessment and pain score

The quality of life was assessed by the Bengali and Hindi version of European Organization for Research and Treatment of Cancer Quality of Life Questionnaire (EORTC QLQ C-30)^10^. It consists of five functional scales, three symptom scales, a global health status/QoL scale, and six single items. Different set of questions are included in the multi-item scale. All scales and single-item measures range in score from 0 to 100. A high score in any functional scale denotes a high/healthy level of functioning, but a high score for a symptom scale/item represents a high level of symptomatology/problems. Functional scales include physical functioning, role functioning, emotional functioning, cognitive functioning, and social functioning. Symptoms scales/items include fatigue, nausea and vomiting, pain, dyspnoea (DY), insomnia, appetite loss, constipation, diarrhoea and financial difficulties. Experience of pain from this debilitating disease was evaluated by the validated Izbicki pain score^9^ having the following components: frequency of pain attacks, a visual analog scale of pain, analgesic medication used, and inability to work. All participants were requested to fill up the EORTC QLQ C30 questionnaire and Izbicki pain score in the hospital before surgery and during each follow up visit.

### Statistical analysis

All the categorical variables were expressed as percentage and the continuous variables were expressed as mean+/-SD as well as median and range value. Comparisons of various domains of QOL and pain scores before and after surgery were performed using ‘paired t test’. Comparison of various domains of QOL and pain scores between alcoholic and non-alcoholic were performed by using ‘individual sample t test’. p≤0.05 was regarded as significant. All calculations were performed with IBM SPSS software, version 26.0.

## RESULTS

One hundred and six patients with CP underwent surgery during the study period. Eight were excluded as five were below 15 years, two had malignancy in final biopsy and one acute illness within four weeks. Thus, 98 patients were included in the present study. Of these, alcohol was aetiology in 42 cases and overall 63 were male (64.3%). In the alcoholic group male:female was 41:1 and it was 22:34 in non-alcoholic group (p=0.00). Mean age at the time of surgery was 34.15 years and it was 39.62 and 30.05 years in alcoholic and non-alcoholic group respectively(p=0.00). There was more smoker in alcoholic group (18 vs. 6, p=0.00). Alcoholic CP also experienced frequent preoperative hospital admission due to abdominal pain (18.14 vs. 9.20, p=0.05). Pancreatic head mass and biliary stricture were distinctly predominant in alcoholic group (18 vs. 4, p=0.00 and 10 vs. 4, p=0.03, respectively). Others demographic and preoperative clinical data in alcoholic and non-alcoholic group are presented in Table 1.

**TABLE 1:** Demographic and preoperative clinical data

	Overall (n=98) (mean+/- sd)	Alcoholic (n=42) (mean+/- sd)	Non-alcoholic (n=56) (mean+/- sd)	p
Age (in years)	34.15+/-11.25	39.62 +/- 10.27	30.05+/- 10.22	0.00
Gender (M:F)	63:35	41:1	22:34	0.00
Weight (in kg)	50.43+/-8.47	51.03+/-8.45	49.98+/-8.53	0.54
Duration of pain (in months)	59.9+/-59.21	54.19+/-64.33	64.18+/-55.28	0.41
Number of prior hospitalization	13.03+/- 22.89	18.14 +/-28.62	9.20+/- 16.68	0.05
Prior surgery/endoscopic therapy	15	4	11	0.25
Smoker	24(24.5%)	18	6	0.00
Endocrine insufficiency	30(30.6%)	11	19	0.50
Exocrine insufficiency	6(6.1%)	3	3	1.00
Preoperative albumin	3.88+/-0.61	3.76+/- 0.60	3.96+/-0.60	0.10
Preoperative hemoglobin	11.60+/-1.84	11.92+/-2.05	11.35+/-1.65	0.12
Preoperative bilirubin	0.73+/-0.43	0.69 +/-0.23	0.77+/- 0.53	0.39
Preoperative ALP	201.92+/-156.15	229.45 +/-177.96	181.27+/-135.58	0.13
Head of pancreas mass	22(22.4)	18	4	0.00
Biliary stricture	14(14.3)	10	4	0.03
Gall stone	1	0	1	1.00
Pseudocyst	2	1	1	1.00
Splenic vein thrombosis	2	1	1	1.00
Pseudoaneurysm	2	1	1	1.00
MPD diameter	7.12+/-2.95	6.47+/- 2.52	7.51+/-3.26	0.09

### Operative data

Operative procedures and data are summarized in Table 2.

**TABLE 2:** Operative procedures and data

	Overall	Alcoholic	Non-alcoholic	p
Type of surgery				
Frey	46(46.9)	28	18	0.00
LPJ	43(43.9)	8	35	
Distal pancreatectomy	5(5.1)	3	2	
Izbicki procedure	3(3.1)	2	1	
Pancreaticogastrostomy	1(1.0)	1	0	
Associated surgery				
Cholecystectomy	9(9.1)	5	4	0.14
Choledochojejunostomy	6(6.1)	4	2	
Hepaticojejunostomy	5(5.1)	3	2	
Reinsertion of CBD in cavity	5(5.1)	4	1	
Cystojejunostomy	3(3.1)	2	1	

Blood loss	161.73+/-121.79	160.71+/-109.62	162.50+/-131.16	0.94
Duration of surgery	248.54+/-43.08	255.52+/-41.788	243.30+/-43.676	0.16
Left sided portal hypertension	21(21.4)	14	7	0.02
Tissue removed in Frey	9.33+/-5.36	10.12+/-6.26	8.11+/-3.34	0.21

The most frequent surgical procedure was Frey procedure (46.9%), closely followed by lateral pancreaticojejunostomy (43.9%). However, Frey procedure was more common in alcoholic group (28 vs. 18) whereas LPJ was distinctly more common in non-alcoholic (35 vs. 8). Most common additional procedure was cholecystectomy. Mean operative time was 248.54 min (+/-43.08) and mean blood loss was 161.73 ml (+/- 121.79). There was no difference in operative time and intraoperative blood loss in alcoholic and non-alcoholic groups (p=0.16 and 0.94 respectively). Left sided portal hypertension was found in 21 patients (21.6%) and it was significantly higher in alcoholic group (14 vs. 7, p=0.02). Mean weight of tissue removed by pancreatic head coring in Frey procedure was 9.33 g (+/-5.36) and it was 10.12(+/-6.26) g and 8.11(+/-3.34) g in alcoholic and non-alcoholic groups, respectively(p=0.21).

### Postoperative data

Mean hospital stay was 9.41 days (+/-2.96). Fifty postoperative complications developed in total 39 patients (39.79 %). Among them, 21 were non-alcoholic and 18 were alcoholic CP (37.5% vs. 42.85%, p=0.67). Twelve major complications (Clavien Dindo 3 or above) developed in 10 patients. There was no postoperative mortality in the present study (Table 3)*.*

**TABLE 3:** Postoperative complications (n=39)

Complication Grade	Complications (n=50)	Details
I	16	Mild wound infection managed in the ward n=10 Biochemical leak (Gr A PF) no intervention n= 6
II	22	Biochemical leak (Gr A PF) requiring discharge with abdominal drain and removed within three weeks, n=9 Post-pancreatectomy haemorrhage requiring blood transfusion, n=3 Chest infection treated with antibiotics and chest physiotherapy, n=2 Delayed gastric emptying (Gr A), managed with prokinetics, n=3 Chyle leak, treated with parenteral nutrition and medium chain triglyceride, n=1 Seizures, controlled with antiepileptic drugs, n=1 Ascites, managed with IV albumin and diuretics, n=1 Hypoglycaemia, n=1 Transfusion reaction, n=1
IIIa	6	Severe wound infection requiring secondary suturing, n=3 Grade B PF, n=3(In two patients percutaneous drain were placed and, in another patient, drain repositioning done)
IIIb	4	Complete wound dehiscence (burst abdomen), requiring secondary suturing under general anaesthesia, n=1 Re-exploration due to post-pancreatectomy haemorrhage (Gr B/C), n=3
IVa	2	Re-exploration due to PPH with single organ dysfunction, n=2 (one developed non-arteritic optic neuritis and other developed acute renal failure)
IVb	0	No multiorgan dysfunction in the study group
V	0	No postoperative death in the study group

Most common postoperative complication was pancreatic leak (n=18). Fifteen patients had biochemical leak that was managed conservatively. Three had grade B postoperative pancreatic fistula. One had persistent drainage beyond three weeks and required repositioning of the drain. Another two developed abdominal collection for which ultrasound guided percutaneous drain was placed. Postoperative pancreatic haemorrhage occurred in eight patients. Among these, Frey procedure was performed in seven and Izbicki procedure in one. Three patients were managed conservatively and five needed re-exploration. The source of bleeding was cored out head cavity in all five. One patient of postoperative haemorrhage developed non-arteritic ischaemic optic neuritis due to severe hypotension. Hospital stay and specific complications were compared, and no difference was found between alcoholic and non-alcoholic groups (Table 4).

**TABLE 4:** Postoperative data comparison

	Overall (n=98)	Alcoholic (n=42)	Non-alcoholic (n=56)	p
Hospital stay (in days)	9.41+/-2.96	9.83+/-3.06	9.09+/-2.87	0.22
Overall complications (no of patients)	39(39.79%)	18(42.85%)	21(37.5%)	0.67
POPF	18(18.36%)			
Grade A	15	9	6	0.21
Grade B	3	2	1	
PPH	8(8.16%)			
Grade A	1	1	0	0.67
Grade B	4	2	2	
Grade C	3	1	2	

POPF=Postoperative pancreatic fistula; PPH=postoperative pancreatic haemorrhage

### Follow-up data

Median follow up was 18 months (6-30). During this period, new onset diabetes mellitus was documented in three patients (two of them underwent Frey procedure, other LPJ). At the end of follow up, 33 patients (33.67%) were diabetic. Of them 23 were on insulin and 10 on oral hypoglycaemic agents. No patient developed new onset exocrine insufficiency and there was no improvement of preoperatively diagnosed exocrine insufficiency. Benign biliary stricture developed in two patients who underwent Frey procedure previously. Choledochojejunostomy was performed in both of them using the same loop that was used for pancreaticojejunostomy. One patient developed incisional hernia and prolene mesh repair was performed. Body weight was not found to be significantly improved after surgery in both the groups (Table 5).

**TABLE 5:** Comparison of body weight

Body weight	Preoperative	At follow up	p
Alcoholic	51.036+/-8.45	51.988+/-8.18	0.269
Non-alcoholic	49.982+/- 8.53	48.893+/-8.33	0.234
Overall	50.434+/-8.47	50.219+/-8.36	0.738

### Quality of life and pain score assessment

Overall quality of life and Izbicki pain score were significantly improved after surgery (Table 6) though 10 patients (10.2%) had incomplete pain relief at their last follow up visit. Among them six were in alcoholic group and four in non-alcoholic.

**TABLE 6:** QOL score and pain score before and after surgery

Parameters	Preoperative Mean±SD	At follow up Mean±SD	p
Global heath score	46.58±18.04	74.31±14.44	0.001
Physical functioning score	67.21±18.84	89.45±16.77	0.001
Role functioning score	62.92±29.26	88.60±15.43	0.001
Emotional functioning score	60.79± 21.67	86.64±14.69	0.001
Cognitive functioning score	63.94±27.98	86.56±18.48	0.002
Social functioning score	63.26±28.31	85.20±18.67	0.001
Fatigue score	46.14±23.84	14.51±17.93	0.002
Nausea and vomiting score	39.45±29.54	12.58±14.76	0.002
Pain score	46.93±30.58	16.83±19.80	0.001
Dyspnoea score	34.69±29.47	9.52±17.91	0.002
Insomnia score	47.27±30.99	17.00±23.56	0.001
Appetite loss score	45.91±30.08	15.30±22.53	0.002
Constipation score	34.69±32.43	13.94±21.89	0.001
Diarrhoea score	33.33±31.74	12.58±18.85	0.001
Financial difficulties score	49.65±32.19	13.26±22.82	0.001
Pain score			
Visual analogue score	85.82±16.74	12.35± 22.64	0.002
Frequency of attack	71.68±24.51	12.24±23.61	0.001
Analgesic medications	18.86±11.36	2.35±5.73	0.001
Inability to work	50.00±21.23	6.38±14.07	0.001
Pain score	56.58±12.12	8.32±14.75	0.001

In 95 patients (96.9%) there was improvement in all parameters of quality of life whereas in three improvement in global health score only. Other parameters of quality of life were not significantly improved in them. When we compared preoperative QOL and pain score between alcoholic and non-alcoholic group, we found financial difficulty to be significantly higher in alcoholic group (p=0.020). During follow up period physical functioning was significantly improved in alcoholic group (p=0.05) but appetite loss was more perceived in non-alcoholic group (p=0.03). Requirement of analgesic medications was more in alcoholic group (p=0.05, Table 7).

**TABLE 7:** QOL score and pain score in alcoholic and non-alcoholic

	Preoperative			During follow up		
	Alcoholic	Non-alcoholic	p	Alcoholic	Non-alcoholic	p
QOL score						
GH Score	45.52±19.64	47.80±16.92	0.540	73.17±14.37	75.14±14.56	0.507
PF Score	63.73±17.95	69.7±19.23	0.123	93.33±10.95	86.67±19.55	0.052
RF Score	61.38±25.39	64.03±31.93	0.660	89.83±14.85	87.71±15.91	0.506
EF Score	59.95±20.51	61.40±22.63	0.747	88.00±13.31	85.67±15.65	0.440
CF Score	61.38±27.24	65.78±28.59	0.445	89.02±18.10	84.79±18.70	0.266
SF Score	59.34±27.90	66.08±28.51	0.248	85.36±14.52	85.08±21.28	0.942
FA Score	47.69±24.24	45.02±23.7	0.588	11.92±16.55	16.37±18.79	0.228
NV Score	42.68±29.59	37.13±29.54	0.362	9.34±9.16	14.91±17.44	0.066
PA Score	50.4±29.92	44.44±31.07	0.344	17.07±20.91	16.66±19.15	0.921
DY Score	35.77±28.27	33.91±30.53	0.760	8.13±17.92	10.52±17.99	0.516
SL Score	43.90±27.32	49.70±33.40	0.363	17.07±23.71	16.95±23.67	0.981
AP Score	48.78±30.81	43.85±29.65	0.427	9.75±17.06	19.29±25.15	0.038
CO Score	34.95±30.68	34.5±33.9	0.946	10.56±20.32	16.37±22.81	0.197
DI Score	39.02±31.53	29.23±31.54	0.133	13.82±19.68	11.69±18.35	0.585
FI Score	58.53±30.53	43.27±32.09	0.020	12.19±22.05	14.03±23.52	0.696
Pain score						
Frequency of attack	76.22±23.68	68.42±24.78	0.121	9.15±17.46	14.47±27.11	0.273
VAS	86.34±16.24	85.44±17.22	0.794	11.46±22.42	12.98±22.98	0.75
Analgesic medications	18.41±10.51	19.18±12.02	0.746	3.68±7.15	1.39±4.26	0.05
Inability to work	52.44±20.77	48.25±21.57	0.338	7.32±15.04	5.70±13.37	0.577
Pain score	58.35±11.75	55.32±12.32	0.224	7.90±14.30	8.36±15.18	0.810

PF=physical functioning; RF=role functioning; EF=emotional functioning; CF=cognitive functioning; SF=social functioning; FA=fatigue; NV=nausea and vomiting; PA=pain; DY=dyspnoea; SL=insomnia; AP=appetite loss; CO=constipation; DI=diarrhoea; FI=financial difficulties

## DISCUSSION

The term ‘tropical pancreatitis’ was coined by Zuidema et al^30^ to describe idiopathic chronic pancreatitis in developing countries. Recent review study by Garg PK^12^ had shown that tropical pancreatitis and idiopathic pancreatitis have distinct clinical course, different genetic pattern and different prognosis. However, idiopathic chronic pancreatitis (or tropical pancreatitis) is considered to be most common variety of chronic pancreatitis in India^13^. In our study we found alcoholic pancreatitis in 42.8% cases and non-alcoholic in 57.2%. Though few cases of pancreas divisum were identified, in absence of genetic analysis we were unable to differentiate between idiopathic chronic pancreatitis and tropical chronic pancreatitis. As a result, non-alcoholic etiology remains the most common etiology in our study group. A research from South India^22^ indicates alcohol intake as a dominant emerging cause of CP and Halder SK et al^15^ had also mentioned alcohol as most common etiology in their study population.

Alcohol intake is not common for woman in Indian culture and as a result we found distinctly male predominant in alcoholic chronic pancreatitis. We also found significantly higher mean age in alcoholic group than non-alcoholic. This reflects several years of chronic alcoholism leads to CP and also idiopathic chronic pancreatitis and tropical chronic pancreatitis both occur in 2^nd^ and 3^rd^ decade^12^. Smoking is itself a risk factor for CP, and also accelerates the progression of CP in alcoholics^19^ ^,^ ^29^. A nationwide study from India by Balakrishnan et al^5^ had identified overall 28.3% smoker in CP patients and 59% of male alcoholics were smokers. In our data smoking was present in 42.8 % (18/42) in alcoholic and only 10.7% (6/56) in non-alcoholic group. This may be explained by male predominance in alcoholic CP group and, also, heavy drinkers are often heavy smokers^5^ ^,^ ^7^. 

Incidence of diabetes (overall 30.6%) was similar to other reported Indian series^5^ ^,^ ^20^ but the incidence of exocrine insufficiency (overall 6.1%) was lower compared to western series^14^ ^,^ ^21^. The possible explanation may be less dietary fat intake by Indian population. During follow up period only three patients developed new onset diabetes and none new onset exocrine insufficiency. The low incidence of new diabetes and new exocrine insufficiency in our study may be due to very short duration of follow-up. Exocrine and endocrine function may deteriorate over time and needs long-term follow-up. However, we were unable to document improvement of body weight. This result can be attributed to very short-term follow up period.

Head of pancreas mass was preoperatively identified in 18 cases (42.8%) in alcoholic group. Bordacahar et al^7^ had found significantly more local inflammation in posterior plane of the pancreas (53% in alcoholic CP and 25% in controls, p=0.05). Biliary stricture was present in 14.3% in our study. Similar result was obtained by in a recent study by Hao et al^16^ where they found common bile duct stricture in 15.8% cases. They had included factors like male, age at onset, smoking, body mass index and morphology of main pancreatic duct to develop a nomogram to predict common bile duct stricture in chronic pancreatitis patients. The clinical profile of alcoholic chronic pancreatitis is also being reflected by nature of surgery performed. Frey procedure was more frequently performed in alcoholic group whereas LPJ was most commonly in non-alcoholic. Interestingly we performed total 46 Frey procedures whereas preoperatively we identified pancreatic head mass only in 22 cases. It may be explained by poor or inadequate preoperative computed tomography scan reporting and/or the mass was less than <4 cm (not being enrolled as pancreatic head mass). This difference was reflected in amount of tissue removed in Frey procedure. Though it is not significantly different in two groups but in alcoholic group it was on higher side (10.12 vs. 8.11 g). In study by Gestic et al^14^ median tissue removed in Frey was 14.8 g (7-78). In their work etiology of CP was alcohol abuse in 95.9% cases.

Several papers^3^ ^,^ ^4^ ^,^ ^25^ had compared Frey with Whipple or Beger procedures and was unable to find any significant difference in terms of pain relief, mortality and long-term morbidity. However, Frey procedure is technically easier and our preferred method. Again, in presence of extrahepatic/left sided portal hypertension the dissection of tunnel of Love beneath the neck of the pancreas, as required in Beger or Whipple procedures is likely to be tedious and risky^21^. In our study we found left sided portal hypertension in 21.4% cases and it was significantly higher in alcoholic group (14 vs. 7, p=0.02). Similar result was also obtained by Bordacahar et al^7^. They found significantly more segmental portal hypertension in alcoholic CP in comparison to non-alcoholic CP (69% vs. 31%, p=0.0069).

There was no perioperative mortality in our study and overall morbidity was present in 39.79% patients which is comparable with other studies^14^ ^,^ ^20^ ^,^ ^21^. Grade A pancreatic fistula or biochemical leak was most common complication in our study. Grade B occurred only in three. Hard texture of pancreatic parenchyma and decreased capacity of secretory function may explain this very low number of clinically relevant postoperative pancreatic fistula.

In our study the EORTC QLQ C-30 questionnaire and Izbicki pain score have been used to assess the quality of life and pain control. Both were well accepted by our patients and required around 22 min (15-40) to complete. There was significant improvement in all domains of QOL after surgery (p<0.005) which was similar to that reported by various authors^4^ ^,^ ^17^ ^,^ ^18^ ^,^ ^24^ ^,^ ^25^. In our study, we found that financial difficulty was significantly higher in alcoholic patients before surgery which might be attributed to alcohol consumption itself. Again, it may reflect the different socioeconomic status in alcoholic patient^9^.

In our study we found 90% to have complete pain relief. Similar result have been reflected by various studies with very long-term follow up^14^ ^,^ ^20^ ^,^ ^21^. Factors like preoperative opiate use, continuous pattern of pain, long duration of pain(>6y), postoperative complications and age ≥38 years were suggested for incomplete pain relief after surgery^20^. Bordacahar et al^7^ had also suggested early surgery and preoperative complete smoking as favourable factor for improved quality of life after surgery in alcoholic CP.

During follow up physical functioning score was significantly improved after surgery in alcoholic group. Physical functioning score involved questions associated with physical work (e.g. any trouble during strenuous exercise or long walk, need to stay in bed during day, etc.). These questions may be not very appropriate for younger patients who are not very accustomed to this type of physical activity. This might be a reason for finding a better physical functioning score in alcoholic group where most of the patients are male and relatively higher age group. Interestingly analgesic requirement was higher in alcoholic group during follow up period. Again, it may reflect the narcotic abuse in alcoholic group. 

However, there are several limitations on this research. First, this study was from a large, tertiary care referral centre. Therefore, it may reflect more severe disease than patients with chronic pancreatitis who are seen in primary care or community gastroenterology practice setting. Second, our median follow up period was only 18 months. Pain control and quality of life both may change in long-term follow up. Third, we have not analyzed the factors for incomplete pain relief in the follow up period.

## CONCLUSION

Alcoholic chronic pancreatitis presents with more local complications than non- alcoholic chronic pancreatitis. Smoking is prevalent in this group and they experienced more hospital admission before surgery. Pancreatic head mass, benign biliary stricture and segmental or left sided portal hypertension are significantly more prominent in alcoholic group in compared to idiopathic or tropical pancreatitis. As a result, resectional surgery is more performed in alcoholic patients. Frey procedure is a safe and well accepted surgery with a very good short-term outcome. Though there is significant difference in some aspects of quality of life (physical functioning and appetite loss) and pain score (analgesics requirement), further long-term data needs to be evaluated for proper conclusion in these parameters. 

## References

[1] Ammann RW (1997). A clinically based classification system for alcoholic chronic pancreatitis summary of an international workshop on chronic pancreatitis. Pancreas.

[2] Ammann RW, Buehler H, Muench R, Freiburghaus AW, Siegenthaler W (1987). Differences in the natural history of idiopathic (nonalcoholic) and alcoholic chronic pancreatitis A comparative long-term study of 287 patients. Pancreas.

[3] Bachmann K, Tomkoetter L, Erbes J, Hofmann B, Reeh M, Perez D, Vashist Y, Bockhorn M, Izbicki JR, Mann O (2014). Beger and Frey procedures for treatment of chronic pancreatitis comparison of outcomes at 16-year follow-up. Journal of the American College of Surgeons.

[4] Bachmann K., Tomkoetter L., Kutup A., Erbes J., Vashist Y., Mann O., Bockhorn M., Izbicki J.R (2013). Is the Whipple procedure harmful for long-term outcome in treatment of chronic pancreatitis? 15-years follow-up comparing the outcome after pylorus-preserving pancreatoduodenectomy and Frey procedure in chronic pancreatitis. Annals of Surgery.

[5] Balakrishnan V, Unnikrishnan AG, Thomas V, Choudhuri G, Veeraraju P, Singh SP, Garg P, Pai CG, Devi RN, Bhasin D, Jayanthi V (2008). Chronic pancreatitis A prospective nationwide study of 1,086 subjects from India. JOP: Journal of the pancreas.

[6] Bassi C (2005). International Study Group on Pancreatic Fistula Definition; Postoperative parcreatic fistula; an international study group (ISGPF) definition. Surgery.

[7] Bordaçahar B, Couvelard A, Vullierme MP, Bucchini L, Sauvanet A, Dokmak S, Ruszniewski P, Lévy P, Rebours V (2018). Predicting the efficacy of surgery for pain relief in patients with alcoholic chronic pancreatitis. Surgery.

[8] Dindo D, Demartines N, Clavien PA (2004). Classification of surgical complications a new proposal with evaluation in a cohort of 6336 patients and results of a survey. Annals of surgery.

[9] Eashwar VA, Umadevi R, Gopalakrishnan S (2020). Alcohol consumption in India-An epidemiological review. Journal of family medicine and primary care.

[10] Fayers P, Bottomley AE, EORTC Quality of Life Group (2002). Quality of life research within the EORTC-the EORTC QLQ-C30. European Journal of Cancer.

[11] Frey CF, Smith GJ (1987). Description and rationale of a new operation for chronic pancreatitis. Pancreas.

[12] Garg PK (2012). Chronic pancreatitis in India and Asia. Current gastroenterology reports.

[13] Garg PK, Tandon RK (2004). Survey on chronic pancreatitis in the Asia-Pacific region. Journal of gastroenterology and hepatology.

[14] Gestic MA, Callejas-Neto F, Chaim EA, Utrini MP, Cazzo E, Pareja JC (2011). Surgical treatment of chronic pancreatitis using Frey's procedure a Brazilian 16-year single-centre experience. Hpb.

[15] Halder SK, Bhattacharjee PK, Bhar P, Das C, Pandey P, Rakshit KP, Pachaury A (2015). A Comparative Study Between Longitudinal Pancreacticojejunostomy v/s Lateral Pancreaticogastrostomy as a Drainage Procedure for Pain Relief in Chronic Pancreatitis Done in a Tertiary Referral Centre of Eastern India. Indian Journal of Surgery.

[16] Hao L, Bi YW, Zhang D, Zeng XP, Xin L, Pan J, Wang D, Ji JT, Du TT, Lin JH, Ye B (2019). Risk Factors and Nomogram for Common Bile Duct Stricture in Chronic Pancreatitis. Journal of clinical gastroenterology.

[17] Izbicki JR, Bloechle C, Broering DC, Knoefel WT, Kuechler T, Broelsch CE (1998). Extended drainage versus resection in surgery for chronic pancreatitis a prospective randomized trial comparing the longitudinal pancreaticojejunostomy combined with local pancreatic head excision with the pylorus-preserving pancreatoduodenectomy. Annals of surgery.

[18] Izbicki JR, Bloechle C, Knoefel WT, Kuechler T, Binmoeller KF, Broelsch CE (1995). Duodenum-preserving resection of the head of the pancreas in chronic pancreatitis A prospective, randomized trial. Annals of surgery.

[19] Luaces-Regueira M, Iglesias-García J, Lindkvist B, Castiñeira-Alvariño M, Nieto-García L, Lariño-Noia J, Domínguez-Muñoz JE (2014). Smoking as a risk factor for complications in chronic pancreatitis. Pancreas.

[20] Negi S, Singh A, Chaudhary A (2010). Pain relief after Frey's procedure for chronic pancreatitis. British Journal of Surgery.

[21] Pessaux P, Kianmanesh R, Regimbeau JM, Sastre B, Delcenserie R, Sielezneff I, Arnaud JP, Sauvanet A (2006). Frey procedure in the treatment of chronic pancreatitis short-term results. Pancreas.

[22] Rajesh G, Girish BN, Panicker S, Balakrishnan V (2015). Time trends in the etiology of chronic pancreatitis in South India. Tropical Gastroenterology.

[23] Srivastava S, Bhatia MS (2013). Quality of life as an outcome measure in the treatment of alcohol dependence. Ind Psychiatry J.

[24] Strate T, Bachmann K, Busch P, Mann O, Schneider C, Bruhn JP, Yekebas E, Kuechler T, Bloechle C, Izbicki JR (2008). Resection vs drainage in treatment of chronic pancreatitis long-term results of a randomized trial. Gastroenterology.

[25] Strate T, Taherpour Z, Bloechle C, Mann O, Bruhn JP, Schneider C, Kuechler T, Yekebas E, Izbicki JR (2005). Long-term follow-up of a randomized trial comparing the Beger and Frey procedures for patients suffering from chronic pancreatitis. Annals of surgery.

[26] Wente MN, Bassi C, Dervenis C, Fingerhut A, Gouma DJ, Izbicki JR, Neoptolemos JP, Padbury RT, Sarr MG, Traverso LW, Yeo CJ (2007). Delayed gastric emptying (DGE) after pancreatic surgery a suggested definition by the International Study Group of Pancreatic Surgery (ISGPS). Surgery.

[27] Wente MN, Veit JA, Bassi C, Dervenis C, Fingerhut A, Gouma DJ, Izbicki JR, Neoptolemos JP, Padbury RT, Sarr MG, Yeo CJ (2007). Postpancreatectomy hemorrhage (PPH)- an international study group of pancreatic surgery (ISGPS) definition. Surgery.

[28] Whitcomb DC (2012). Genetics of alcoholic and non-alcoholic pancreatitis. Current opinion in gastroenterology.

[29] Yadav D, Slivka A, Sherman S, Hawes RH, Anderson MA, Burton FR, Brand RE, Lewis MD, Gardner TB, Gelrud A, DiSario J (2010). Smoking is underrecognized as a risk factor for chronic pancreatitis. Pancreatology.

[30] Zuidema PJ (1959). Cirrhosis and disseminated calcification of the pancreas in patients with mal-nutrition. Tropical and geographical medicine.

